# An overview of the influence and design of biomaterial of the intraocular implant of the posterior capsule opacification

**Published:** 2018

**Authors:** Razvan Vladimir Nanu, Emil Ungureanu, Sinziana Luminita Instrate, Alexandra Vrapciu, Roxana Cozubas, Laura Carstocea, Liliana Mary Voinea, Radu Ciuluvica

**Affiliations:** * “Sf Ioan” Emergency Hospital, Bucharest, Romania; ** Ophthalmology Department, University Emergency Hospital; “Carol Davila” University of Medicine and Pharmacy, Bucharest, Romania; *** ”Grigore Alexandrescu” Emergency Hospital for Children, Bucharest, Romania; **** PhD Student, “Carol Davila” University of Medicine and Pharmacy, Bucharest, Romania; ***** Anatomy Department, “Carol Davila” University of Medicine and Pharmacy, Bucharest, Romania

**Keywords:** cataract surgery, intraocular implant, posterior capsule opacification

## Abstract

Posterior capsule opacification remains till nowadays one of the most hypothetical problems concerning the cataract surgery. When it comes in preventing PCO, this complication is made in multiple ways that concern, along with the surgery steps, the choice for the biomaterial of the intraocular implant lens. The concern of influence of the type of the used material (hydro-phob/ hydro-philic), of the design of the implant (1-piece IOL = monobloc vs. 3 - piece IOL – multipiece) and with the design at the edge, they all have been considered in multiple studies. This article performs a synthesis of those studies and establishes conclusions regarding possible choices.

**Abbreviations:** PCO = Posterior capsule opacification, IOL = intraocular lens; LEC = lens epithelial cells

## Introduction

Cataract is the second cause of legal blindness (which means visual acuity < 0.05 at presentation) in Europe, after age related macular degeneration. In certain regions of Central and East Europe cataract is the main cause of legal blindness [**1**], with over 30% of the discovered cases [**2**]. Therefore, the surgery of cataract is, of course, one of the most frequently performed surgical procedures in the world.

The surgery of cataract has evolved and constantly improved when it comes to the technique, but also in regarding the material of the artificial lens and design. The first intraocular implant ever made (Ridley I) was manufactured in the year of 1949 from PMMA (poly-methyl-methacrylate). The first lenses made from PMMA were implanted after the surgery was performed, with an extra-capsular techniques, which determined high astigmatism because of the large incisions. In the 70’s, Charles Kelman was the one who introduced cataract surgery through phaco-emulsification. This technique was the opening road in smaller incisions and creating a reason for bio-material to appear, to be researched and remodeled, determining the new apparition for the lenses that folded, made from silicone material, hydrogel, or acrylic lenses [**3**,**4**].

From the point of view of design of the lens, but as well as the material, we can consider multiple choices of implants, the designs evolving continually, the target now in research being consisted from the possibility that is obtain through implanting the lens through a minimal incision. The intracellular inflammatory response is as mild as possible for us to prevent the PCO and lens epithelial cells proliferation. The implants are widely variable when it comes to chemical formula, water content, refraction index, but also, in case of the design. Here, it presents different shapes of the part of the optic, the haptics, and different at edge profiles. The target of these variations is to allow a minimum de-centration and dislocation, but also a very small rate of PCO and to reduce optical aberrations [**5**].

The research literature was realized through Pubmed, and the article allows a synthesis of the material and designs available and their influence over the PCO rate. 

## Posterior capsule opacification 

Trauma made during the surgery will determine the breakdown between the components of the aqueous-blood barrier. The consequence is an outflow of proteins and macrophage cells to the surgery area, which will induce an early postoperative inflammation. LEC proliferation will determine the accumulation of the cells over the posterior capsulae of the lens, determining PCO, but also at the level of the anterior capsulae of the lens, making ACO. The source of PCO is from the equatorial epithelial cells which can make metaplasia, and which allows their proliferation and, after that, migration. PCO is now the most constant complication in regarding the cataract surgery, notwithstanding with the efforts for creating a better material and better designs [**6**]. Treatment of PCO consists of neodymium: YAG laser (Nd:YAG) capsulotomy, but this is not without complications of their own: implant deterioration, spikes of intraocular pressure, with possible secondary glaucoma; rarely cystoid macular oedema and retinal detachment [**7**].

PCO physiopathology is a multifactorial one, but also individual elements are separated – the surgical technique, the IOL material, the IOL design; all the influence coming from this parts is difficult to separate in clinical practice.

In **Table 1** are showed the PCO rates that drag to the necessity of capsulotomy, made for different materials and for different designs. Increased rates in PCO and also in Nd:YAG capsulotomy have been linked to the acrylic hydrophilic, but also with the PMMA materials [**8**-**21**]. 

**Table 1:** PCO linked to the Nd:YAG capsulotomy rates for articles 2008 and 2018

Authors	Study design	Follow-up	Number of eyes	IOL model	Characteristics	PCO rate	Nd:YAG capsulotomy rate
Hancox [**8**]	Prospective randomized, contralateral	24 month	36	AcrySof SN 60AT (Alcon)	1 piece acrylic hydrophobic	8.83%	-
			36	AF-1 YA-60BB (Hoya)	acrylic hydrophobic	32.45%	-
Hayashi [**9**]	Prospective randomized, contralateral	12 month	45	Acrysof MA60AC (Alcon)	3 piece, optic rotund, acrylic hydrophobic	-	2.19%
			45	AR40e (AMO)	3 piece, optic rotund, acrylic hydrophobic	-	2.20%
Kohnen [**10**]	Prospective randomized, contralateral	37 month	139	CeeOn Edge 911A (AMO) vs. Acrysof MA60BM (Alcon)	Silicone versus acrylic hydrophobic		2.1% vs. 2.1%
			108	AMO vs. PhacoFlex SI40NB	Square edge versus round edge		5.7% vs. 17%
		2 years	60	BL27 (B&L)	acrylic hydrophobic		42%
Kugelberg [**11**]	Prospective randomized			AcrySof SA60AT (Alcon)	acrylic hydrophobic		10%
Boureau [**12**]	Retrospective	2.9 years	250	AcrySof SA60AT (Alcon)	1 piece, acrylic hydrophobic	13.6%	12%
			254	AR40e (AMO)	3 piece, acrylic hydrophobic	26.8%	25.2%
			263	XL Stabil (Zeiss)	1 piece, acrylic IOL hydro-philic	52.9%	50%
Ronbeck [**13**]	Prospective randomized,	5 years	54	809C (Pharmacia)	Round edges IOL, PMMA	100%	54%
			48	SI-40NB (AMO)	Round edges IOL, PMMA	12%	29%
			50	Acrysof MA60 BM (Alcon)	Square edges, acrylic IOL hydrophobic	18%	8%
Vock [**14**]	Retrospective	10 years	98	Acrysof MA60BM	3 piece, square edges, acrylic hydrophobic	9%	42%
			44	SI-30NB/SI-40NB	3 piece, round edges, silicone	39%	18%
Gathier [**15**]	Bilateral, retrospective	2 years	160	AcrySof ReSTOR (Alcon)	acrylic hydrophobic		8.8%
			152	AcriLisa (Zeiss IOL)	acrylic hydrophilic with hydrophob in surface		37.2%
Iwase [**16**]	Prospective randomized, also contralateral	2 years	63	Acry Sof SA60 AT	1 piece, square edges, acrylic hydrophobic		2%
			63	Meridian HP60 M (B& L)	1 piece, double square edges, 1 piece, acrylic hydrophilic		13%
Vasavada [**17**]	Prospective randomized, contralateral	3 years	66	AcrySof IQ SN60WF (Alcon) vs. C-flex 570 C (Rayner)	acrylic hydro-phobic vs. acrylic hydro-philic		0% vs. 12.9%
			62	AcrySofIQ SN60WF (Alcon) vs. Akreos AdaptAO (B&L)	acrylic hydro-phobic vs. acrylic hydro-philic		0% vs. 16.1%
Chang [**18**]	Prospective randomized	5-7 years	40	Acrysof SA60AT (Alcon)	1 piece, acrylic hydro-phobic		22%
			40	Sensar AR40e (AMO)	3 piece, acrylic hydro-phobic		10%
Bourdiol Ducasse [**19**]	Retrospective	2-3 years	126	Acrysof SN60WF (Alcon)	1 piece, square edges, acrylic hydrophobic		10.3%
			89	Akreos AO-MI 60 (B&L)	1 piece, square edges, acrylic hydrophilic		36%
			85	Hoya YA-60 BB (Hoya)	3 piece, square edges, acrylic hydrophobic		24.9%
Fong [**20**]	Prospective, cohort	3 years	101	AcrySof SA60AT (Alcon)	1 piece, square edges, acrylic hydrophobic	34.4%	
			67	MA50BM (Alcon)	3 piece, square edges, acrylic hydrophobic	50.80%	
			156	Sensar AR 40 e (AMO)	3 piece, round edges, acrylic hydro-phobic	38.5%	
			101	Akreos AdaptAO (B& L) / Quatrix (Croma)	Square edges, acrylic hydro-philic	64.4%	
Ursell et al. [**21**]	Retrospective	3 years	13.329	AcrySof SA60 AT (Alcon)	1 piece, square edges, acrylic hydrophobic	4.7%	2.4%
			19.025	Non-Acrysof	hydrophobic	6.3%	4.4%
			19.808	Non-Acrysof	hydrophobic	14.8%	10.9%

In a this retrospective study [**12**], Boureau did compared the incidence of the laser Nd:YAG capsulotomy made for the different IOL types: 12% for Alcon (SA 60 AT), 25% were performed for AMO (AR 40 e) and 51% for Zeiss (XL – Stabi). Gauthier did reported [**15**] a smaller rate - 8.8% for Nd:YAG capsulotomy for Acry Sof Re STOR (brand Alcon) respectively 37% for Acri LISA (Zeiss). Bourdiol Ducasse have reported in this study [**19**] a lower statistically significant rate for the capsulotomy with Acry Sof lenses compared to Hoya or Akreos (Bausch & Lomb).

All those results can be determined by the hydrophobic IOL’s adherence to collagen membranes [**22**], with a better apposition between the artificial lens and the lenses posterior capsule, with a very small space through which the LEC are possible not being able to migrate. Also, another study has reported [**23**] that hydro-philic IOL’s could promote the so called proliferation, but also the migration of LEC from the lens equator into the visual central area.

Concerning the comparison of hydrophobic vs. silicon IOL’s, studies have shown controversial results. Some studies [**10**,**14**] also compared PCO in regarding with the rates of Nd:YAG capsulotomy, comparing square edges 3-piece silicon and IOL hydro-phobic and acrylic. After 3 years of this study, the results were similar in rates of PCO, but without being statistically significant in differences. Another study [**13**] did compare PCO together with the rate of Nd:YAG capsulotomy between the silicone 3 – piece round edges vs. acrylic hydrophobic square edges lens. The results did confirmed that, in this case, silicone lenses were able to determine the inhibition of PCO in regarding a longer period of time (for over 4-5 years) [**24**]. 

Independent of the IOL material, it has been concluded that regarding the IOL design, the square edge reduces the PCO rate. A systematic study [**25**] has shown that the PCO rate was significantly lower for square edges vs. the IOLs with round edges independently from the IOL material. This has been given to the fact that an edge with a square posterior represents a barrier for the LEC in migration. However, we did find also authors who would take the consideration that the IOL in square edge efficiency it is still correlated together with the material of the IOL in regarding PCO and the rate of Nd:YAG capsulotomy [**18**].

We consider obvious that the primary space for the LEC intrusion could be the interior of a haptic loop, so this is translated into pointing out the very much importance in designing the haptic in PCO. It was proven that a reduced angulation of the haptic, a “C” loop, and a thin perpendicular haptic insertion over the optic have been factors which were associated with a very small and reduced rate of LEC migration, PCO and Nd:YAG capsulotomy [**22**].

We cite also some of the studies [**26**,**27**] with different conclusions between the 1-piece vs. 3-piece IOL’s influence over the PCO rate. One study [**26**] has determined a reduced rate of the PCO for 1-piece implants, but in other studies [**27**] we did not find any statistically different rates of the PCO. 

Studies concerning the size of the optic zone [**28**] did shown a good association between large optical areas and lower rates of PCO. 

## Conclusions 

We consider that there is an evident influence between the material of the IOL and the rate of the PCO. Most of the studies enumerated in our review have shown a smaller rate of PCO for acrylic hydrophobic IOL’s in comparison to PMMA and also acrylic hydro-philic IOL’S, while we saw the evidence in regarding the silicone IOL’s that is not that clear. There are also design factors to be considered, most importantly the posterior edge design. Posterior square edges have determined a reduced rate of PCO compared to round edges. In the studies presented, the best results were achieved for acrylic hydro phobic or silicone lenses, with the posterior square edges. Other factors, such as the design of the haptic zone, the optical area design, and aspherical surface of the optic part might also present a pretty small influence over the rate of the PCO. 

**Acknowledgements**

All authors have equal contribution.
